# Impairment of intermediate somatosensory function in corticobasal syndrome

**DOI:** 10.1038/s41598-020-67991-7

**Published:** 2020-07-07

**Authors:** Kana Matsuda, Masayuki Satoh, Ken-ichi Tabei, Yukito Ueda, Akira Taniguchi, Keita Matsuura, Masaru Asahi, Yuichiro Ii, Atsushi Niwa, Hidekazu Tomimoto

**Affiliations:** 1 Department of Neurology, Mie University Graduate School of Medicine, Tsu, Japan; 2 Department of Rehabilitation, Mie University Graduate School of Medicine, Tsu, Japan; 3 Department of Dementia Prevention and Therapeutics, Mie University Graduate School of Medicine, 2-174 Edobashi, Tsu, Mie 514-8507 Japan

**Keywords:** Neurodegeneration, Neurodegenerative diseases

## Abstract

Corticobasal syndrome (CBS) is characterized by unilateral atrophy of the brain. New diagnostic criteria for CBS include intermediate somatosensory dysfunction. Here, we aimed to carefully examine intermediate somatosensory function to identify tests which can assess impairment in CBS patients. Using voxel-based morphometry (VBM), we also aimed to show the anatomical bases of these impairments. Subjects included 14 patients diagnosed with CBS and 14 patients with Parkinson's disease (PD). Patients were evaluated using intermediate somatosensory tests and neuropsychological assessments. VBM was used to analyze differences in gray matter volumes between CBS and PD patients. In the PD group, no tests showed a significant difference between the dominant-side onset and the non-dominant-side onset. In the CBS group, all tests showed worse scores on the affected side. For detecting intermediate somatosensory dysfunction in CBS, two tests are recommended: tactile object naming and 2-point discrimination. VBM analysis showed that the volume of the left post- and pre-central gyrus, and both sides of the supplementary motor area were significantly decreased in the CBS group compared to the PD group. Although CBS remains untreatable, early and correct diagnosis is possible by performing close examination of intermediate somatosensory function.

## Introduction

Somatosensory functions are subdivided into two main groups^1–4^: the elementary somatosensory functions consisting of light touch, pain, thermal sensation, joint position sense, and vibration sense; and the intermediate somatosensory functions including 2-point discrimination, tactile localization, weight, texture, and shape perception. The intermediate somatosensory functions are thought to be the integration of various tactile information in the cerebral cortex, and their dysfunction is localized to the postcentral gyrus. One of the symptoms caused by intermediate somatosensory disorders is clumsy limb, otherwise known as limb kinetic apraxia.

Corticobasal degeneration (CBD) was considered a primary motor disorder characterized by asymmetrical rigidity with apraxia and various other features, including cortical sensory loss, alien limb behavior, myoclonus and dystonia^5–8^. Neuropathologically, it has been reported that CBD is associated with tau-positive intracellular inclusions, cortical ballooned neurons, frontoparietal neuronal loss and gliosis, and nigral and basal ganglia degeneration^9^. Recently, it has become clear that these classic clinical features are observed only in subsets of patients, and are associated with many other pathologies, such as Alzheimer’s disease (AD) and progressive supranuclear palsy (PSP). The background pathologies of patients diagnosed with CBD are varied^10^. In 1999, Boeve et al. examined 13 cases clinically diagnosed with CBD and concluded that pathological diagnosis is necessary to correctly diagnose CBD^11^. Moreover, the classic neuropathology of CBD is also found in patients with progressive aphasia or frontotemporal dementia, so it is difficult to use the term CBD as a unified clinicopathological entity. This variability in pathology has led a number of researchers to propose the term “corticobasal syndrome” (CBS) as a clinical designation. In 2001, Cordato et al. first used the name CBS for a clinical diagnosis^12^. It has since been pointed out that there are considerable differences between the clinical and pathological diagnoses, i.e. CBS versus CBD, respectively.

On the other hand, from a different perspective, it became clear that CBD also has various clinical features. In 2013, Armstrong reviewed the clinical features of 267 patients who were pathologically diagnosed with CBD and found 4 CBD phenotypes emerged: CBS, frontal behavioral-spatial syndrome (FBS), nonfluent/agrammatic variant of primary progressive aphasia (naPPA), and progressive supranuclear palsy syndrome (PSPS)^13^. CBS presents with many atypical cases, and several clinical diagnostic criteria have been reported^14–17^. Among the new diagnostic criteria, “cortical sensory loss” or intermediate somatosensory dysfunction was present in approximately one quarter of cases pathologically diagnosed with CBS^18^. However, despite being one of the diagnostic criteria for CBS, there have been no reports that thoroughly evaluated the impairment of intermediate somatosensory function in CBS patients.

In this study, we closely examined intermediate somatosensory function in CBS and PD patients, and compared these results. Using voxel-based morphometry (VBM), we also investigated the relationship between grey matter volume and intermediate somatosensory dysfunction.

## Subjects and methods

### Subjects

The subjects included 14 patients diagnosed with CBS based on published criteria^18^ at Mie University Hospital from April 2013 to August 2017 (CBS group). In 12 of the participants, their right side was affected. As a control group, 14 patients diagnosed with PD based on published criteria^19^ at Mie University Hospital from November 2016 to March 2017 were also recruited (PD group). The H&Y scale for the PD group was 1–2. In 10 of these patients, the right side was dominant-side onset. PD was chosen as the control because some tests of intermediate somatosensory function require hand or finger movements and active touch which might be affected by extrapyramidal dysfunction in both CBS and PD.

### Methods

Intermediate somatosensory function and neuropsychological tests were performed at nearly the same time as the MRI. All procedures followed the Clinical Study Guidelines of the Ethics Committee of Mie University Hospital and were approved by the internal review board (Registration number: 2450). A complete description of all procedures was provided to the patients, and written informed consent was obtained from them or their caregivers.

### Intermediate somatosensory function

Function was assessed based on reported literature^1–4^. For 2-point discrimination, the examiner placed a pair of plastic needles from a slide caliper on the index and the little finger pad of the patients, whose eyes were closed, and asked them to report the number of needles they were touched with, “one” or “two”. Forty-two trials were performed for each hand. For tactile localization, the examiner used a pen to touch a point from the second to fifth finger on the right or left hand of the patients, whose eyes were closed, then asked them to indicate the location of the point using the first finger of the same hand. Twenty-four trials were performed for each hand. For texture perception, 5 wooden plates of an identical size and shape were prepared on which 1 of 5 different textures (rough or fine sandpaper, plastic, paper, vinyl, or paper towel) was mounted. The patients palpated 2 textures serially with their eyes closed, then they were asked to perform ‘same-different’ discrimination. Twenty-five trials were tested for each hand. For tactile object discrimination, 12 daily objects (fork, bottle opener, clip, key, pin, dry cell battery, eraser, rubber band, matchstick, nut, bottle cap, and clothes pin) were used. The patients palpated 2 of them serially with their eyes closed, then they were asked whether these objects were the same in 20 trials for each hand. For tactile object naming, the patients were asked to name a single manipulated object in 12 trials for each hand. For shape perception, the examiner wrote a number or hiragana (Japanese cursive characters) on the patient’s hand, and asked them to report what letter was written in 16 trials for each hand. For weight perception, the patients were asked to arrange objects in the correct weight order with either their left or right hand. The objects were 6 plastic cases of equal size, shape, and texture weighing 50, 60, 70, 80, 90, or 100 g.

### Neuropsychological assessments

Function was assessed based on reported literature^19,24,27^. The Mini-Mental State Examination (MMSE)^21^ and Japanese Raven’s Coloured Progressive Matrices (RCPM)^22^ were used to quantify cognitive function. In addition to an overall score, the RCPM task also measures performance time which reflects the psychomotor speed of the participants, and this was also assessed. Memory was evaluated using the Rivermead Behavioral Memory Test (RBMT)^23^. Visuospatial ability was assessed using the Mie Constructional Apraxia Scale (MCAS)^24^. Frontal lobe function was assessed using two tasks: word fluency (WF) and the Trail Making Test (TMT)-A/-B^25^. The WF test consists of category and letter domains. In the categorical WF task (WF-Category), participants were asked to name as many animals as possible in 1 min. In the letter WF task (WF-Letter), participants were asked to name as many objects as possible in 1 min beginning with each of the following four phonemes: *ka, sa, ta,* and *te*^26^. The average scores for these four phonemes were used for statistical analyses. Apraxia was evaluated using the praxis test of the Western Aphasia Battery (WAB). Skilled movement was assessed by imitation of hand movement (I–II ring and I–III–IV ring) and manipulation of daily objects (turn a page, open and close the lid of a plastic bottle, fasten/remove a button, put on/remove gloves, and grab an item).

### MRI acquisition

As described previously by Tabei et al.^27^, for voxel based morphometry (VBM), the parameters used for 3D-T1-weighted imaging were as follows: TR, shortest; TE, 15 ms; flip angle, 90°; FOV, 230 × 230 mm; matrix size, 256 × 256; slice thickness, 1.1 mm; acquisition time, 6 min 20 s.

### MRI analysis

As shown by Tabei et al.^27^, MRI data were analyzed using SPM12 (Wellcome Trust Centre for Neuroimaging, University College London, London, United Kingdom) implemented in MATLAB R2012a (MathWorks, Natick, MA, USA). In the pre-processing phase, images were set to match the anterior to posterior commissure line using an automated MATLAB script. The images were then visually inspected to check for possible scan issues such as field distortion and movement artifacts. Reoriented images were corrected for intensity inhomogeneity and segmented into gray matter (GM), white matter (WM), cerebrospinal fluid, and other tissues outside the brain using SPM12 tissue probability maps. The images were registered to the East Asian Brains International Consortium for Brain Mapping space template via affine regularization. We created a population-specific template using the SPM12 DARTEL template procedure to directly compare data between the CBS group and the PD group. We investigated group differences in GM volume, as well as the relationship between neuropsychological assessment results and GM at the whole-brain level. High-dimensional DARTEL was used to create non-linear, modulated-normalized GM and WM images, which were smoothed using a Gaussian kernel of 8 mm FWHM (full-width at half-maximum). For whole-brain and multiple regression analyses, we assessed the statistical significance at a voxel threshold of *p* < 0.005 (uncorrected), within contiguous clusters of at least 20 voxels. We obtained both Montreal Neurological Institute (MNI) and Talairach coordinates to detect the anatomical regions of the clusters. We used a transform from Matthew Brett1 to convert MNI coordinates to Talairach coordinates, and Talairach Client 2.4.3 (Lancaster et al. 2000) was used to identify the anatomical regions corresponding to the Talairach coordinates^27^. The initial voxel threshold was set to 0.001 uncorrected. Clusters were considered as significant when falling below a cluster-corrected p (FWE) = 0.05.

### Statistical analyses

The analysis was carried out based on previous reports^19,27^. Differences in demographic variables and results from the neuropsychological assessment between the CBS and PD groups, and in the impairment of intermediate somatosensory function between unaffected and affected sides were analyzed using independent *t* tests for continuous data, chi-square tests for dichotomous data, and Mann–Whitney U tests for nonparametric data. Differences of *p* < 0.05 were considered statistically significant. Statistical analyses were performed using IBM SPSS Statistics software version 20 (IBM Corp., Armonk, NY, USA).

## Results

### Participant characteristics and neuropsychological assessments

Patients in the CBS and PD groups showed no significant differences in age (*p* = 0.13) or disease duration (*p* = 0.11). The following neuropsychological assessments were significantly lower in the CBS group than in the PD group (Table 1): MMSE (*p* = 0.02), RCPM (*p* = 0.01), Standard Profile Score (SPS; *p* = 0.01) and Screening Score (SS; *p* = 0.02) of RBMT, TMT-A (*p* = 0.01), and WF-Category (*p* < 0.01). Compared to the RCPM scores of age-matched standards (26.9 ± 5.396), the intellectual function of all patients in both groups was preserved within normal limits.

**Table1:** Characteristics and neuropsychological assessments of the patients.

	CBS (n = 14)	PD (n = 14)	*P* value
**Demographics**			
Age, years, mean (SD)	71.9 (7.01)	66.6 (8.71)	0.137
Education, years, mean (SD)	10.8 (1.74)	13.0 (1.75)	0.014*
Disease duration, years, mean (SD)	2.71 (1.31)	4.28 (3.53)	0.11
Male sex, no. (%)	7 (50)	6 (42.8)	0.717
**Neuropsychological test**			
MMSE	24.7 (4.53)	28.0 (3.03)	0.027*
RCPM			
Score	23.2 (6.31)	28.7 (6.32)	0.016*
Time, s	769.5 (618.8)	440.0 (201.2)	0.131
RBMT			
SPS	14.9 (3.77)	19.1 (5.03)	0.019*
SS	6.07 (2.39)	8.71 (3.04)	0.025*
TMT			
A, s	271.4 (152.3)	202.3 (219.5)	0.012*
B, s	409.5 (321.8)	237.3 (158.2)	0.111
WF,/min			
Animal	8.46 (3.04)	13.7 (4.41)	0.002*
Letters	4.53 (2.10)	6.28 (2.97)	0.094
Construction			
MCAS	2.69 (1.66)	2.25 (1.76)	0.713

*MCAS* Mie Constructional Ability Scale, *MMSE* Mini-Mental State Examination, *RBMT* Rivermead Behavior Memory Test (SPS: Standard profile score, SS: Screening Score) , *RCPM* Raven’s Colored Progressive Matrices, *s* seconds, *TMT* Trail-Making Test, *WF* word fluency.

*Statistically significant.

### Intermediate somatosensory functions

**Figure 1:**
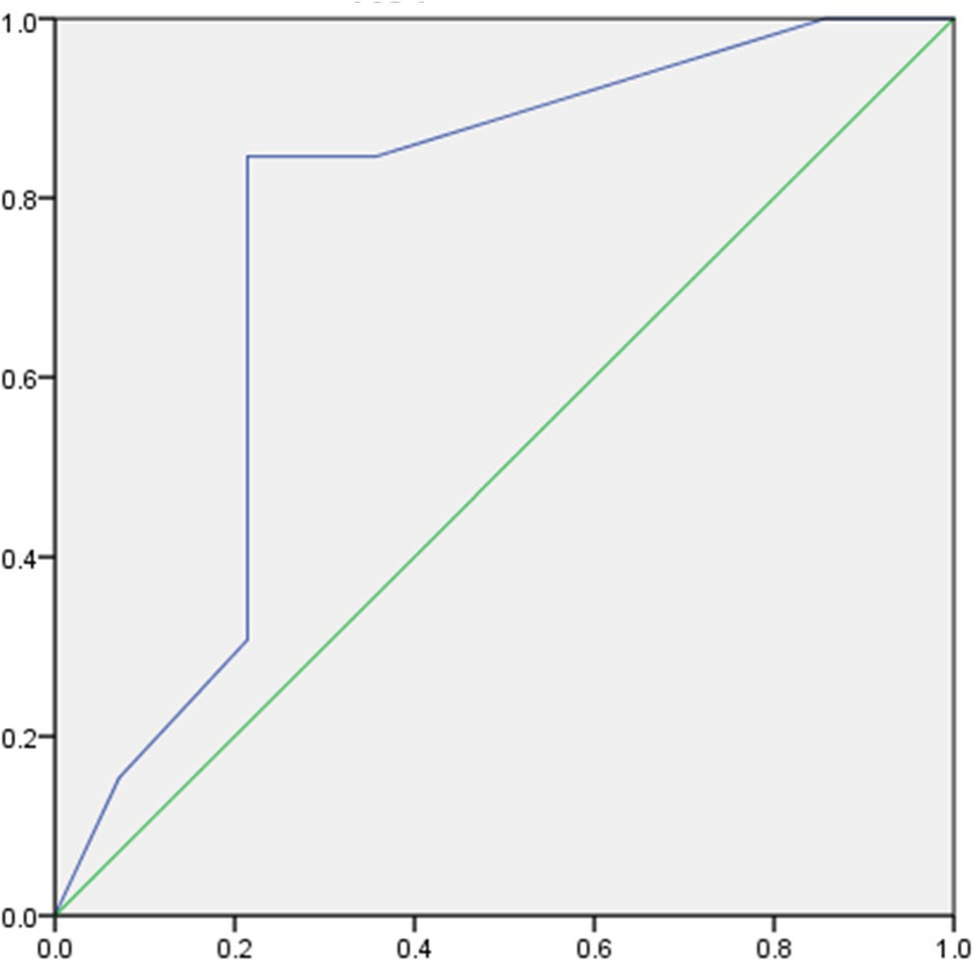
ROC curve for object naming (affected side). Cut off: 8.5/12, sensitivity: 84.6%. Specificity: 78.6%, AUC: 0.764.

In the PD group, no tests showed a significant difference between the dominant-side onset and the non-dominant-side onset. In the CBS group, all tests showed worse scores on the affected side. Five tests (tactile localization, shape perception, tactile object naming, texture perception, weight perception) showed significantly worse scores on the affected side (Table 2). All patients in the PD group could perform all tests. In the CBS group, all patients were able to perform the graphesthesia and texture perception tests, and 85.7% (12/14) were able to perform tactile object naming, tactile object discrimination, 2-point discrimination, and weight perception (6/7; Table 3). There was a significant difference between the unaffected side and the affected side in 41.6% (5/12) of the patients in tactile object naming and 33.3% (4/12) with 2-point discrimination (Table 4). Considering the number of patients who could perform the tests, and those who showed significant differences between the unaffected and the affected side, we can preliminarily conclude that tactile object naming and 2-point discrimination are good methods to evaluate intermediate somatosensory dysfunction in CBS patients. For these two tests, we conducted receiver operating characteristic (ROC) analysis. Given that the 2-point discrimination provides an actual value and since the area under the curve (AUC) was low, only the results from tactile object naming are presented. In ROC analysis, the tactile object naming score had an AUC of 0.76 (95% CI 0.57–0.95). This analysis determined that the cut-off value was 8.5 for the score on the affected side (sensitivity of 84.6%, specificity of 78.6%; Fig. 1).

**Table 2:** Results of somatosensory test in the CBS and PD groups.

Tests	CBS			PD		
	Unaffected side	Affected side	*P* value	Non-dominant side onset	Dominant side onset	*P* value
Tactile localization (/24)	23.1 (2.10)	21.2 (2.18)	0.01*	23.2 (1.85)	23.1 (2.17)	0.33
Shape perception (/16)	9.14 (4.01)	7.78 (4.22)	0.01*	14.0 (2.25)	13.3 (2.64)	0.2
Tactile object naming (/12)	7.92 (1.75)	7.07 (2.28)	0.02*	9.78 (2.48)	9.57 (2.76)	0.58
Tactile object discrimination (/20)	18.7 (2.05)	18.2 (2.92)	0.11	20	20	n.s
Texture perception (/25)	22.5 (2.21)	20.0 (3.89)	< 0.01*	23.6 (1.33)	22.8 (1.29)	0.81
2-point discrimination (cm)	5.32 (1.65)	6.64 (2.37)	0.05	3.50 (1.47)	3.94 (1.59)	0.49
Weight perception (g)	15.0 (8.36)	26.6 (8.75)	< 0.01*	10.1 (0.57)	13.0 (4.42)	0.17

In the PD group, none of the items showed a significant difference between the dominant-side onset and the non-dominant side onset. In the CBS group, all items showed a worse score in the affected side and 5/7 items showed a statistically significant worse score in the affected side (*).

*n.s.* not significant.

**Table 3:** Number of cases who could perform the tests in the CBS group.

Tests	n (%)
Tactile localization	9 (64.2%)
Shape perception	14 (100%)
Tactile object naming	12 (85.7%)
Tactile object discrimination	12 (85.7%)
Texture perception	14 (100%)
2-point discrimination	12 (85.7%)
Weight perception	6/7 (85.7%)

All patients in the PD group could perform all tests.

**Table 4:** Number of cases significantly different between unaffected and affected sides in the CBS group.

Tests	n (%)
Tactile localization	1/9 (11.1%)
Shape perception	1/14 (7.1%)
Tactile object naming	5/12 (41.6%)
Tactile object discrimination	0/12 (0%)
Texture perception	1/14 (7.1%)
2-point discrimination	4/12 (33.3%)
Weight perception	0/7 (0%)

The denominator represents the number of cases that could be tested, and the numerator represents the number of cases that were significantly impaired.

### MRI assessments

**Figure 2:**
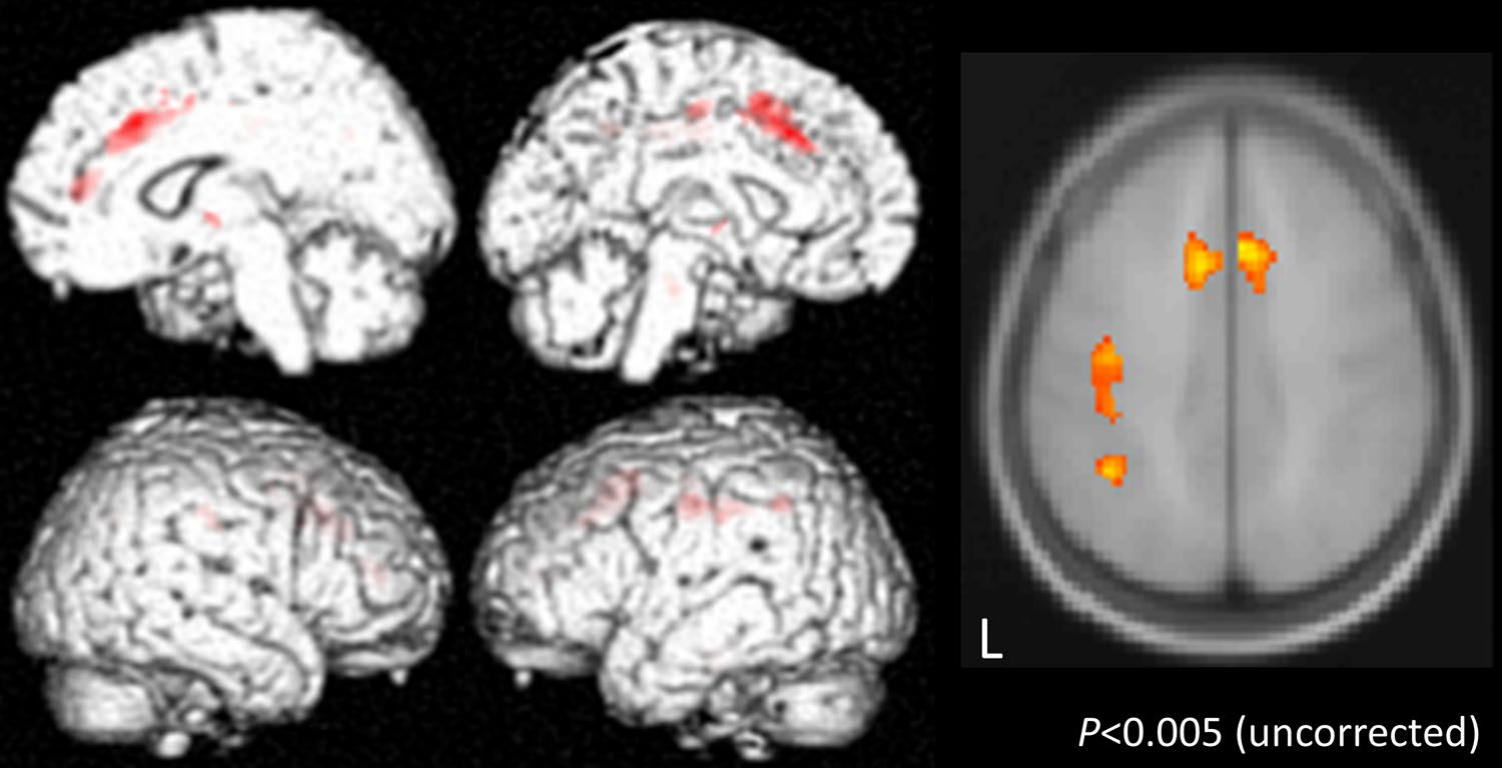
Voxel-based morphometry (VBM) comparing the CBS and PD groups. VBM analysis was performed on PD and CBS patients who had symptoms on the right side. The following area volumes were significantly decreased in the CBS group compared to the PD group: the left postcentral gyrus and precentral gyrus, and both sides of the supplementary motor area.

VBM analysis was performed for patients in the PD and CBS groups who had symptoms on the right side. The following area volumes were significantly reduced in the CBS group compared to the PD group: the left postcentral gyrus and precentral gyrus, and on both sides of the supplementary motor area (Fig. 2).

## Discussion

This study investigated the impairment of intermediate somatosensory function in CBS patients and PD patients. Despite being one of the diagnostic criteria of CBS, there have been no reports that closely evaluated the impairment of intermediate somatosensory function in CBS patients. Thus, we examined these functions in detail to identify which tests may be the most useful for assessing CBS patients. Furthermore, using VBM, we identified differences in GM volume between CBS and PD patients.

In regard to cognitive function, the CBS group showed lower scores on each test than the PD group. Cognitive dysfunction is included as one of the diagnostic criteria for CBS, although the degree of cognitive dysfunction in our cases was mild compared with previous studies^28–30^. For intellectual function, the RCPM scores were within the average range for the age of the patients. Moreover, all patients were able to speak, read, write and listen in everyday life, and there were no symptoms of aphasia or severe memory dysfunction. We thus assume that the results from the intermediate somatosensory tests in the present study are reliable.

In the CBS group, all tests of intermediate somatosensory function showed worse scores on the affected side. In the PD group, no significant difference was observed between the dominant-side onset and the non-dominant-side onset. Thus, intermediate somatosensory dysfunction is a characteristic finding of CBS, which is congruent with results of previous studies^18,31,32^. The evaluation of intermediate somatosensory function is important because it was included in the diagnostic criteria of CBS. Many of the studies published on CBS reported the presence or absence of intermediate somatosensory dysfunction, but the authors did not specify which tests were used to investigate function^33,34^. To our knowledge, only a few reports have described the tests that were used: position sense^35,36^, stereognosis^37^, and graphesthesia^38^. The present study suggests that two tests may effectively detect intermediate somatosensory dysfunction in CBS and are thus recommended: tactile object naming and 2-point discrimination. Only a few reports have described using these tests; however, they did not indicate the number of tasks nor the evaluation method^39,40^. Given that 2-point discrimination was evaluated using actual values and the AUC in the ROC analysis was low, only tactile object naming was utilized. The analysis identified that the cut-off value of tactile object naming was 8.5 for the score on the affected side. Tactile object naming is thought to be suitable for detecting intermediate somatosensory dysfunction in CBS because the task requires complex hand/finger movements depending on the shape, weight, and texture of each object, and the function of the pre- and post-central gyrus might be required more than in other tests. Thus, we conclude that close evaluation of intermediate somatosensory function is indispensable for determining whether a patient fulfils the diagnostic criteria of CBS or not.

Results of the VBM analysis showed that the volume of the left post- and pre-central gyrus, and bilateral supplementary motor area, were significantly decreased in the CBS group compared to the PD group. A typical MRI image from a CBS patient shows asymmetric atrophy near the central sulcus^5,6^. Previous reports on VBM in CBS patients have shown that the primary motor areas and parietal operculum^41^ were significantly decreased compared with normal age-matched controls, and these findings have been thought to be related to the occurrence of “clumsy limb” (limb kinetic apraxia). Three reports have shown the relationship between CBD and the supplementary motor area^42–44^. Two of these studies used VBM to demonstrate that the supplementary motor area and premotor cortex were associated with the occurrence of limb apraxia^42^ and ideomotor apraxia^43^. The other report investigated the neural and cognitive bases of upper limb apraxia in CBD using resting [18F]-fluorodeoxyglucose PET scanning^44^. This study found hypometabolism at the superior parietal lobule and supplementary motor area. Thus, all of these reports describe the relationship between the supplementary motor area and the occurrence of apraxia. Also, the supplementary motor area has a mild somatotopy from the anterior to posterior regions, and the upper limbs are said to have somatotopy at the anterior region^45^. The CBS patients in this study also has atrophy at the anterior part of the supplementary motor area, which may be associated with clumsy hand.

**Figure 3:**
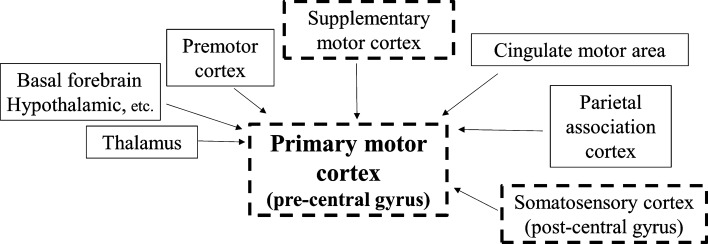
Input to the primary motor cortex. The primary motor cortex receives inputs from higher centers such as the premotor cortex and supplementary motor cortex, the somatosensory cortex, the parietal association cortex, and subcortical organization such as the thalamus and the basal forebrain. The dotted squares show that whose volume were significantly decreased in the CBS group.

The supplementary motor area, along with the somatosensory cortex, sends information to the primary motor cortex (Fig. 3). Thus, in CBS patients, degeneration of the post- and pre-central gyrus, and the supplementary motor area, might be involved in the occurrence of clumsy limb. In the present study, nine out of twelve CBS patients who underwent VBM analysis had limb apraxia in the right hand. While the GM volumes of the bilateral supplementary motor areas were significantly reduced, the symptoms of clumsy limb only appeared on the right hand. This difference in the lateralization of limb apraxia might be caused by the dysfunction derived from the degree of atrophy in the post- and pre-central gyrus.

Here, we identified which tests of intermediate somatosensory function are likely to be impaired in CBS patients, and also determined the cut-off points which can be used to assess the presence or absence of impairment. Structural and functional imaging approaches provide some aid in the diagnosis of CBS but have low-content validity. None of the currently available tau-PET ligands is suitable for detecting straight filament 4repeat tau disease in clinical routine. Research on imaging, blood, and cerebrospinal fluid biomarkers is ongoing, but not yet available in clinical routines^46^. Therefore, intermediate somatosensory function is important as a neurological finding. Although a treatment for CBS is not yet available, early and correct diagnosis might be possible by performing a close examination of intermediate somatosensory function which may lead to new approaches to appropriate treatment strategies.

## Limitations

There were some limitations of this study that should be noted. First, this study included a relatively small sample size. However, most previous reports^47–50^ had almost the same number of patients or fewer than in the present study. Second, for the VBM, this study only included cases in which the right side was affected. It is unknown whether the same result would be obtained by lesions on the left side. Third and lastly, we could not perform pathological examination on the subjects. However, atrophy of the supplementary motor area has also been suggested as an imaging biomarker for prenatal diagnosis of CBS^51^, which is consistent with this study. These issues should be investigated in future studies.

## References

[1] Satoh M, Terada S, Onouchi K, Takeda K, Kuzuhara S (2002). Somatosensory and skin temperature disturbances caused by infarction of the postcentral gyrus. J. Neurol..

[2] Dijkerman HC, De Haan EHF (2007). Somatosensory processes subserving perception and action. Behav. Brain. Sci..

[3] Takeda K (1991). Sensorimotor disturbances in patients with the postcentralgyrus. Adv. Neurol. Sci..

[4] Takeda K (2000). The rostrocaudal gradient for somatosensory perception in the human postcentral gyrus. J. Neurol. Neurosurg. Psychiatry.

[5] Mathew R, Bak TH, Hodges JR (2011). Diagnostic criteria for corticobasal syndrome: A comparative study. J. Neurol. Neurosurg. Psychiatry Epub..

[6] Belfor N, Amici S, Boxer A, Kramer J, Tenpini M, Rosen H (2006). Clinical and neuropsychological features of corticobasal degeneration. Mech. Ageing Dev..

[7] Südmeyer M (2012). longitudinal deformation-based morphometry reveals spatio-temporal dynamics of brain volume changes in patients with corticobasal syndrome. PLoS One Epub..

[8] Jütten K (2014). Neuropsychological and brain volume differences in patients with left- and right-beginning corticobasal syndrome. PLoS One Epub..

[9] Schneider J, Watts R, Gearing M, Brewer R, Mirra S (1997). Corticobasal degeneration: Neuropathologic and clinical heterogeneity. Neurology..

[10] Caselli RJ, Jack CR (1992). Asymmetric cortical degeneration syndromes: A proposed clinical classification. Arch. Neurol..

[11] Boeve BF, Maraganore DM, Parisi JE, Ahlskog JE (1999). Pathologic heterogeneity in clinically diagnosed corticobasal degeneration. Neurology.

[12] Cordato N, Halliday G, McCann H, Davies L, Williamson P, Fulham M (2001). Corticobasal syndrome with tau pathology. Mov. Disord..

[13] Armstrong MJ (2013). Criteria for the diagnosis of corticobasal degeneration. Neurology.

[14] Boeve BF, Lang AE, Litvan I (2003). Corticobasal degeneration and its relationship to progressive supranuclear palsy and frontotemporal dementia. Ann. Neurol..

[15] Aiba I (2012). Corticobasal syndrome: Recent advances and future directions. Brain Nerve..

[16] Shimohata T, Nishizawa M (2013). Clinical aspects of corticobasal syndrome. Brain Nerve..

[17] Shelley BP, Hodges JR, Kipps CM, Xuereb JH, Bak TH (2009). Is the pathology of corticobasal syndrome predictable in life?. Mov Disord..

[18] Armstrong MJ, Litvan I, Lang AE, Bak TH, Bhatia KP (2013). Criteria for the diagnosis of corticobasal degeneration. Neurology.

[19] Ueda Y (2016). Neuropsychological features of microbleeds and cortical microinfarct detected by high resolution magnetic resonance imaging. J. Alzheimer’s Dis..

[20] Postuma RB (2015). MDS clinical diagnostic criteria for Parkinson's disease. Mov. Disord..

[21] Folstein M, Folstein S (1975). Mini-Mental state'—a practical method for grading the cognitive state of patients for the clinician. J. Psychiatr. Res..

[22] Raven J (1947). Coloured Progressive Matrices Sets A, AB, B. Manual Sections A and 2.

[23] Wilson B, Cockburn J, Baddeley A (1985). The Rivermead Behavioural Memory Test.

[24] Satoh M (2016). Improved necker cube drawing-based assessment battery for constructional apraxia: The Mie Constructional Apraxia Scale (MCAS). Dement. Geriatr. Cogn. Dis. Extra..

[25] Partington J, Leiter R (1949). Partington's pathways test. Psychol. Serv. Center J..

[26] Dohi N, Iwaya T, Kayamori R (1992). Seishin-kinou Hyouka, the Evaluation of Mental Function.

[27] Tabei K (2018). Cognitive function and brain atrophy predict non-pharmacological efficacy in dementia: The Mihama-Kiho Scan Project2. Front. Aging Neurosci..

[28] Graham NL, Bak TH, Hodges JR (2003). Corticobasal degeneration as a cognitive disorder. Mov. Disord..

[29] Koss S (2010). Numerosity impairment in corticobasal syndrome. Neuropsychology..

[30] Lee S (2011). Clinicopathological correlations in corticobasal degeneration. Ann. Neurol..

[31] Southi N, Honan CA, Hodges JR, Piguet O, Kumfor F (2019). Reduced capacity for empathy in corticobasal syndrome and its impact on carer burden. Int. J. Geriatr. Psycgiatry.

[32] Niccolini F (2018). Disease-related patterns of in vivo pathology in Corticobasal syndrome. Eur. J. Nucl. Med. Mol. Imaging.

[33] Hu W (2009). Alzheimer’s disease and corticobasal degeneration presenting as corticobasal syndrome. Mov. Disord..

[34] Boeve BF (2011). The multiple phenotypes of corticobasal syndrome and corticobasal degeneration: Implications for further study. J. Mol. Neurosci..

[35] Rinne JO, Lee MS, Thompson PD, Marsden CD (1994). Corticobasal degeneration. A clinical study of 36 cases. Brain.

[36] Riley DE (1990). Cortical-basal ganglionic degeneration. Neurology.

[37] Constantinides VC, Paraskevas GP, Efthymiopoulou E, Stefanis L, Kapaki E (2019). Clinical, neuropsychological and imaging characteristics of Alzheimer’s disease patients presenting as corticobasal syndrome. J. Neurol. Sci..

[38] Deutschländer AB, Ross OA, Dickson DW, Wszolek Z (2017). Atypical parkinsonian syndromes: A general neurologist’s perspective. Eur J Neurol Epub..

[39] Wadia PM, Lang AE (2007). The many faces of corticobasal degeneration. Parkinson. Relat. Disord..

[40] Mahapatra RK, Edwards MJ, Schott JM, Bhatia KP (2004). Corticobasal degeneration. Lancet Neurol..

[41] Borroni B (2008). White matter changes in corticobasal degeneration syndrome and correlation with limb apraxia. Arch. Neurol..

[42] Whitwell JL (2010). Imaging correlates of pathology in corticobasal syndrome. Neurology.

[43] Huey E (2009). Association of ideomotor apraxia with frontal gray matter volume loss in corticobasal syndrome. Arch. Neurol..

[44] Peigneux P (2001). Neural and cognitive bases of upper limb apraxia in corticobasal degeneration. Neurology.

[45] Fontaine D, Capelle L, Duffau H (2002). Somatotopy of the supplementary motor area: Evidence from correlation of the extent of surgical resection with the clinical patterns of deficit. Neurosurgery..

[46] Svenningsson P (2019). Corticobasal degeneration: Advances in clinicopathology and biomarkers. Curr. Opin. Neurol..

[47] Cho H (2017). 18F-AV-1451 binds to motor-related subcortical gray and white matter in corticobasal syndrome. Neurology.

[48] Smith R (2017). In vivo retention of 18F-AV-1451 in corticobasal syndrome. Neurology.

[49] Kikuchi A (2016). In vivo visualization of tau deposits in corticobasal syndrome by 18FTHK5351 PET. Neurology.

[50] Soliveri P (1999). Cognitive and magnetic resonance imaging aspects of corticobasal degeneration and progressive supranuclear palsy. Neurology.

[51] Franziska A, Sandrine B, Rodrigo M, Jane N, Matthias LS (2017). Disentangling the neural correlates of corticobasal syndrome and corticobasal degeneration with systematic and quantitative ALE meta-analyses. NPJ Parkinson's Dis..

